# Development of an item list to assess the forgotten joint concept in shoulder patients

**DOI:** 10.1186/s12891-015-0520-7

**Published:** 2015-03-24

**Authors:** Johannes M Giesinger, Nicolas Kesterke, David F Hamilton, Bernhard Holzner, Bernhard Jost, Karlmeinrad Giesinger

**Affiliations:** Department of Psychosocial Research and Epidemiology, The Netherlands Cancer Institute, Plesmanlaan 121, 1066 Amsterdam, CX The Netherlands; Department of Orthopaedics and Traumatology, Kantonsspital St. Gallen, Rorschacherstrasse 95, 9000 St. Gallen, CH Switzerland; Department of Orthopaedic Surgery, University of Edinburgh, 49 Little France Crescent, Edinburgh, EH16 4SA UK; Department of Psychiatry and Psychotherapy, Innsbruck Medical University, Anichstr. 35, A-6020 Innsbruck, Austria

**Keywords:** Shoulder, Forgotten joint score, Questionnaire, Patient-reported outcome, Item bank, Outcome measure

## Abstract

**Background:**

To generate an item list for the assessment of joint awareness in shoulder patients and to collect patient feedback on the comprehensibility of the items and the forgotten joint concept.

**Methods:**

Item content was generated on the basis of literature search and expert ratings following a stepwise refinement procedure, including final evaluation by an international expert board (n = 12) including members with various professional backgrounds. Items were translated from English to German and evaluated in 30 German-speaking shoulder patients in Switzerland and 30 shoulder patients in the UK.

**Results:**

Literature search identified 45 questionnaires covering 805 issues potentially relevant for the assessment of joint awareness. Stepwise item selection resulted in 97 items to be evaluated by the international expert board leaving 70 items for collecting patient feedback. The majority of patients indicated that the introductory text explaining the forgotten joint concept was easy or very easy to understand (79.3%) and that the items were clear (91.4%).

**Conclusion:**

We developed a list of 70 questions for the assessment of joint awareness in shoulder patients and obtained positive patient feedback for these. In a next step, we will administer the items to a large international patient sample to obtain data for psychometric analysis and development of a measurement model, which is the basis for creation of computer-adaptive assessments or static short-forms.

## Background

Patient-reported outcomes (PROs) are key parameters in the evaluation of many orthopedic interventions. A number of well-validated self-report questionnaires are available to assess the patient’s health status from a generic overall health or joint-specific perspective. Such questionnaires are commonly used in orthopedic studies as primary or secondary outcome measures and in joint registers for quality assurance purposes (e.g., Sweden [1], Denmark [2], UK [3], and Switzerland [4]).

In 2012 a novel PRO instrument, the Forgotten Joint Score - 12 (FJS-12), was introduced for use in hip and knee surgery to evaluate the patients’ perspective of the outcome of their treatment. This questionnaire is designed to determine the patients’ awareness of their hip or knee. We believe that the ability to ‘forget’ about a joint in everyday life is the optimal result of any treatment [5]. The ‘forgotten joint’ concept naturally subsumes various domains, as it requires the absence of pain, substantial functional limitations and stiffness etc. This makes this construct especially relevant for treatment evaluation in patient groups with good to excellent outcome and for mid- to long-term assessment. The FJS-12 has been validated in hip and knee patients and provides higher discriminatory power and responsiveness and is less prone to a ceiling effect compared with other traditional PRO questionnaires [5,6].

This paper examines the extension of the forgotten joint concept to the assessment of shoulder patients. The new shoulder measure aims to cover an extensive measurement range and to be applicable for all shoulder pathologies (e.g., fracture, osteoarthritis, instability, rotator cuff tears) and their conservative or operative treatment (e.g., osteosynthesis, prostheses, stabilizing, or rotator cuff surgery).

The shoulder version of the Forgotten Joint Score relies on Item Response Theory (IRT) to develop an item bank, i.e., a set of items and their measurement characteristics. Within an IRT framework, psychometric item characteristics can be explored in detail, e.g., to discover whether items have different measurement characteristics in different patient populations (differential item functioning (DIF)). This is important, as DIF can be a source of substantial measurement bias.

Additionally, IRT-based item banks allow the creation of static short forms with questions relevant to a specific patient population, or tailoring of the questions even to the level of the individual patient (computer-adaptive testing; CAT). Within CAT the patient is asked to complete a starting item that allows calculation of an initial score estimate. Based on this score estimate, an algorithm selects the next question from the item bank to maximize measurement precision. The procedure stops when a desired measurement precision is reached or a maximum number of items have been asked.

The quality of an item bank relies substantially on conceptual considerations, on the measured domain, and on the process of item content development [7] that precedes IRT modeling. Qualitative patient feedback collected from the target group is essential for guaranteeing content validity of an item bank and is also recommended by the US Food and Drug Administration (FDA) in their PRO guidelines [8].

The work presented in this paper entails the qualitative groundwork of the development of an item bank to assess the forgotten concept in shoulder patients. This comprises the definition of the forgotten joint concept, the generation of a list of shoulder issues to be covered by items, item generation, expert evaluation of the items, and collection of patient feedback on the items. This will be followed by a large-scale study to develop an IRT measurement model and determine the measurement characteristics of the items. Development of the item list followed the approach of the European Organisation for Research and Treatment of Cancer (EORTC) Quality of Life Group that is currently developing item banks for a range of patient-reported outcome domains relevant to cancer patients [9-11].

## Methods

### Definition of the concept

Based on initial work on the forgotten joint concept in hip and knee patients [6,12,13], we decided to assess the frequency of joint awareness during activities of daily living as a joint-specific PRO measure. Joint awareness is simply defined as any unintended perception of a joint. This may include strong sensations like pain, but also subtler feelings like mild stiffness or discomfort, subjective dysfunction, or just awareness without pain or discomfort. Generally, joint awareness comes with a negative connotation because perfectly healthy, well-functioning joints do not cause joint awareness in daily life – and are essentially considered to be ‘forgotten.’

### Response format

The response format was adopted from the FJS-12 hip and FJS-12 knee to guarantee consistency between different measures of the forgotten joint concept. It comprises five response categories: “never – almost never – seldom – sometimes – mostly”. In the FJS-12 validation study [5] the response format had been tested in a pilot sample and was revised, both to provide better discrimination in patients with good to excellent outcome and to reduce ceiling effects.

### Literature search and issue development

To generate issues (i.e., item content) with potential relevance for the assessment of shoulder joint awareness during activities of daily living, we performed a literature search on questionnaires used in outcome studies in shoulder patients. The literature search was informed by work by Suk et al. [14] on orthopedic outcome measures and by screening PubMed (http://www.pubmed.org), using “shoulder” and “questionnaire” as search terms. In detail, the search term was: *(“shoulder”[MeSH Terms] OR “shoulder”[All Fields]) AND (“questionnaires”[MeSH Terms] OR “questionnaires”[All Fields] OR “questionnaire”[All Fields]).*

From this search, we set up a list of all the issues covered by the items in the identified questionnaires and rated the issues on their relevance for the assessment of the forgotten joint concept as defined above.

### Operationalization and item selection

The issues collected in the literature search were operationalized into items and refined in several steps, each including three independent expert reviews of each item and a harmonized review based on discussion in cases of disagreement. The individual steps were as follows:

- Development of item wording: the issues rated as relevant were phrased to refer to the frequency of joint awareness and to fit the response categories described earlier. This was done by three raters in close collaboration.
- Removal of duplicates and redundant items: all items rated as duplicates, redundant, or strongly overlapping another item in content were deleted from the item list. Each reviewer did this based on an individual ad-hoc categorization to deal with the large number of items. This allowed easier identification of similar items.
- Evaluation of item difficulty: to guarantee sufficient coverage of the measurement range (i.e., measurement of low, moderate, or high levels of joint awareness), the reviewers estimated all item difficulties in three categories. This aimed at identifying a potentially imbalanced distribution of item difficulties, allowing the development of further items for respective difficulty levels.

### Item evaluation by expert board

The refined item list was sent to an international expert board for a final evaluation. This board comprised 12 members: three orthopedic surgeons, five psychologists, two language professionals, one physiotherapist and one statistician (six Austrians, three Germans, two British, and one Swiss). Items were assessed for clarity, relevance for the forgotten joint concept, and content overlap. Additionally, the introductory text for the items was assessed for clarity and for how adequately it reflected the forgotten joint concept. Experts were encouraged to suggest further items to guarantee content coverage.

### Item translation

The item list was developed in English and then translated to German for collecting patient feedback for both language versions. The translation procedure followed a standardized forward-backward approach as suggested by the American Association of Orthopedic Surgeons (AAOS) Outcomes Committee [15] and the EORTC Quality of Life Group [16]. This means, that the English version was independently translated to German by two native German speakers who were fluent in English. The two translations were harmonized based on discussion between the two translators. To check for ambiguity introduced by translation, the German version was back-translated to English by two native English speakers (fluent in German) and harmonized again. The harmonized version was compared with the original English version and checked for differences.

### Linguistic validation and patient feedback

To investigate the appropriateness of the item list developed in the previous steps, shoulder patients treated at the orthopedics department of the Kantonsspital St. Gallen (Switzerland) and the New Royal Infirmary of Edinburgh (Scotland, UK) provided feedback in the form of debriefing questionnaires after completing the item list. In this qualitative assessment we investigated whether the introductory text and the items were difficult to understand, whether items were intrusive, and whether patients found it difficult to report on their joint awareness. Additionally, patients were encouraged to raise further potentially relevant issues and to make general comments on the item list. Written informed consent was obtained from individual patients for their anonymized data to be used for research purposes. Ethical approval was obtained from the Scotland Research Ethics Committee (UK) and the Ethics Committee of the Canton of St. Gallen (Switzerland).

## Results

### Literature search and issue list

**Figure 1:**
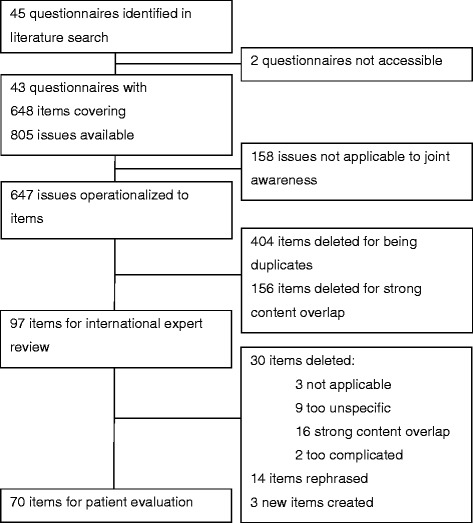
**Flow-chart on item list development.**

Our literature search identified 45 questionnaires assessing PROs in shoulder patients (see section on list of questionnaires below). Full text versions of 43 questionnaires with a total of 648 items were available. In case of instruments including clinician assessment and patient-report, we included both with the exception of objective measurements. The 648 items covered 805 issues potentially relevant to shoulder patients, as several items included two or more issues (e.g., “I have difficulty opening, holding, pushing, or pressing [e.g., triggers, levers, heavy doors]”. All issues were assessed by three raters (two orthopedic surgeons, one psychologist) concerning their relevance for the assessment of the forgotten joint concept. All three raters agreed on in/exclusion of 68.5% of the issues, whereas for 31.5%, only two raters agreed. After a consensus discussion, 158 issues were dropped and 647 remained in the list. Please see Figure 1 for an overview of the item development process.

#### List of questionnaires identified in the literature search

1. Wolfgang criteria tor rating results of rotator cuff surgical repair
2. Shoulder pain score
3. Single Assessment Numeric Evaluation (SANE) rating
4. Watson shoulder score
5. Melbourne Instability Shoulder Scale (MISS)
6. Walch Duplay shoulder instability score
7. Shoulder function assessment (SFA) scale
8. Swanson shoulder score
9. Upper extremity Functional index (UEFI)
10. Upper extremity functional limitation scale
11. Rowe shoulder score
12. Rockwood score for sternoclavicular joint arthritis
13. Shoulder Pain and Disability Index (SPADI)
14. American Shoulder and Elbow Surgeons (ASES) shoulder assessment
15. Upper Extremity Function Scale for Upper Extremity Disorders
16. McGinnis and Denton rating scale for scapula fractures
17. Western Ontario Osteoarthritis of the Shoulder (WOOS) Index
18. Simple shoulder test (SST)
19. Penn shoulder scale (PSS)
20. Hospital of the University of Pennsylvania shoulder score
21. Shoulder rating questionnaire
22. Subjective Shoulder Rating Scale (SSRS)
23. Western Ontario Shoulder Instability Index (WOSI)
24. Western Ontario Rotator Cuff Index (WORC)
25. Modified Rowe shoulder score
26. Imatani acromioclavicular separation evaluation system
27. DASH - Disabilities of the Arm, Shoulder and Hand
28. Rotator Cuff Quality of Life measure (RC-QOL)
29. Herscovici shoulder scale
30. Harryman rotator cuff functional assessment
31. Upper Limp Functional Index (ULFI)
32. UCLA end-result score
33. The Japanese Orthopedic Association Shoulder 36 1.3
34. Oxford instability score
35. Shoulder instability questionnaire
36. Oxford Shoulder Score (OSS)
37. Darrow Score for acromioclavicular separation
38. United Kingdom Shoulder Disability Questionnaire (SDQ-UK)
39. Flexilevel scale of Shoulder Function (Flex-SF)
40. Constant-Murley shoulder score
41. Shoulder activity rating scale
42. UCLA shoulder rating score
43. Thorling subjective rating for subacromial decompression
* Shoulder severity Index (SSI)
* Athletic shoulder outcome scoring system

*questionnaires not accessible.

### Operationalization and item selection

The retained issues were operationalized into items by one orthopedic surgeon and one psychologist. The items were phrased to assess frequency of joint awareness and to fit the previously-mentioned response categories. In a next step, the 647 items were checked for duplicates by one rater, which substantially reduced the number to 243. Further assessment of redundancy (strong content overlap, e.g., taking off a pullover/putting clothes over your head) was done by three raters (two orthopedic surgeons, one psychologist). For 71.4% of the items, all three raters agreed on the redundancy rating. After a consensus discussion (focusing especially on which item of a group of similar items was best retained), 97 items remained in the item list.

The difficulty of these 97 items was rated by three orthopedic surgeons. The difficulty ratings were identical (all three raters) for 38.2% of the items; for 57.7% of items, the raters chose adjacent categories. Aggregated ratings were as follows: 18 low-difficulty items (e.g., turning a key), 52 moderate-difficulty items (e.g., placing a jacket on a hanger), and 27 high-difficulty items (e.g., throwing a ball).

### Item evaluation by expert board

The 97 items were reviewed by the international expert board. On the basis of these reviews, we deleted 30 items, added 2 new items and rephrased 14 items for clarity. Reasons for deletion were the following: 3 items described activities applicable only to a few patients (e.g., playing golf), 9 items were too non-specific (e.g., playing a musical instrument, engaging in sexual activity), 16 were considered to still have strong content overlap with other items, and two were rated as too cumbersome (e.g., recreational activities in which you take some force or impact through your hand).

In parallel we performed an update of the literature search (in August 2013), which identified one additional questionnaire [17] from which one new issue was added to our item list after being made more specific and passing expert evaluation.

After this elaborate procedure, 70 items remained for translation into German and subsequent use in collecting patient feedback.

### Item evaluation by patients

The German and English items were evaluated by 30 shoulder patients at the Kantonsspital St. Gallen and 30 shoulder patients at the New Royal Infirmary of Edinburgh. The patients (63% male; mean age 46.6y, SD 18.2) consisted of a heterogeneous convenience sample of common shoulder problems with surgery for rotator cuff pathologies (43%) and joint replacement (26%) being the most common types of surgery.

Patients rated the understandability of the introduction as follows: very easy 27.6%, easy 51.7%, neither easy nor difficult 19.0%, very difficult 1.7%. Answering questions on shoulder awareness was rated as slightly more difficult: very easy 20.0%, easy 41.7%, neither easy nor difficult 30.0%, difficult 5.0%, and very difficult 3.3%.

We did not observe a statistically significant difference between countries with regard to difficulties with answering questions on joint awareness. For understandability of the introduction we found a statistical trend (p = 0.07) suggesting that the German version was slightly easier to understand.

91.4% of patients reported no item to be difficult to understand and 96.6% considered none of the items to be intrusive. No item was rated as difficult by more than one patient, whereas two patients considered the item on wiping the bottom as intrusive.

Based on these findings we did not exclude items from the item list and did not make any amendments to the introductory text.

Patient feedback did not result in creation of further items, as the suggested activities were either already covered by very similar items (e.g., washing and drying dishes or lying on one’s back) or described uncommon activities (e.g., holding a wind instrument). For details on patient comments, see section on patients’ comments on item list. The final issue list to be used for large-scale data collection in a next step and IRT analysis is given in the section on the final issue list.

#### Patients’ comments on item list

General comments:

- Answers depend on whether or not taking pain medication
- Don’t or can’t do sports (four patients)
- Many activities I don’t do

Suggested further activities:

- Putting on a cap
- Putting on ear rings
- Putting on a scarf
- Washing and dry pans and dishes
- Playing rugby
- Pushing a door handle down
- Holding a wind instrument
- Lifting arm above breast height
- Lying on the back
- Cutting or peeling vegetables

#### Final issue list to be used for large-scale data collection and IRT analysis

1. Taking off pullover
2. Brushing teeth
3. Using telephone
4. Putting on trousers
5. Watching television
6. Washing face
7. Using knife and fork
8. Light recreational activities
9. Ironing clothes
10. Applying deodorant
11. Reaching overhead to high shelf
12. Threading belt through trousers
13. Blow drying hair
14. Working on computer
15. Washing armpits
16. Taking a shower
17. Pulling chair out from table
18. Changing bed linen
19. Lying on affected side
20. Going for a walk
21. Swimming
22. Drying back with towel
23. Playing sports involving overhead serve
24. Taking exercise classes
25. Cleaning windows
26. Carrying small children
27. Carrying heavy suitcase
28. Using banister when climbing stairs
29. Do-it-yourself jobs around the house
30. Folding clothes
31. Closing zip of jacket
32. Turning steering wheel in car
33. Reaching for seat belt in car
34. Putting on coat or jacket
35. Taking off coat or jacket
36. Putting heavy object on shelf at shoulder level
37. Putting light object on shelf above head
38. Drinking from large glass
39. Unfastening belt
40. Holding overhead rail
41. Doing hair
42. Light garden work
43. Wiping bottom
44. Riding bicycle
45. Sitting for an hour
46. Buttoning up shirt/blouse
47. Putting on shoes
48. Handwriting
49. Working overhead >2 minutes
50. Washing hair
51. Turning key
52. Reaching for backseat in car
53. Pushing open heavy door
54. Pulling out of back pocket
55. Hanging jacket on coat-hanger
56. Opening tight jam jar
57. Leaning on elbow
58. Getting on bus/train
59. Performing sudden movement
60. Push-ups
61. Clapping hands
62. Carrying shopping bag
63. Swinging arms when walking
64. Getting out of car
65. Throwing ball
66. Light housework
67. Heavy housework
68. Before falling asleep
69. Resting
70. Scratching between shoulder blades

## Discussion

This article describes in detail the qualitative aspects of item bank development, the foundations of any PRO measure. In our study we comprehensively reviewed the literature for shoulder questionnaires and developed a list of 70 items as the basis for an item bank. All items focus on patients’ joint awareness of the affected shoulder in activities of daily living. The items were rated with regard to various pre-defined criteria and then refined in a stepwise process by international experts. To include patient input in the process, we had patients subsequently evaluate the items (30 patients each for the English and German versions).

The study demonstrates that patients found it an easy task to rate awareness of their shoulder joint in everyday life and that patients also found the introductory text that explained this novel construct easy to understand. Because the item list evaluated by patients was comprehensive, additional activities suggested for inclusion by patients did not present a relevant extension of content coverage or of the item pool’s measurement range.

The presented 70 items constitute a solid basis from which to create an IRT-based item bank from which targeted short-forms or computer-adaptive assessments may be created.

Development of PRO instruments based on an IRT framework has gained interest in various fields of medical research [9,18-20] in the last two decades. However, IRT-based outcome measures are still not commonly available, especially in the surgical specialties, despite the known advantages concerning measurement precision and the possibility of tailoring item sets to individual patients or patient groups to reduce the patient burden introduced by extensive static questionnaires. In the orthopedic field, IRT has been applied in only a few studies [21-24]. To date the computer-adaptive pilot version of the FJS-12 [12] for hip and knee assessment is, to the best of our knowledge, the first computer-adaptive joint-specific measure [25-27]. However, because of its limited item bank, this pilot version represents primarily a proof of principle rather than an elaborate CAT instrument with an extensive item bank.

The largest initiative on the development of item banks for physical and psychosocial health outcomes is the US-led PROMIS group. To date, PROMIS has released a substantial number of item banks, including an item bank for the assessment of physical functioning in all types of patient groups [28-30]. This item bank has recently been extended to further reduce floor and ceiling effects, thus allowing precise measurements at both extremes of the physical function continuum [31]. An important focus of research has been the extent of differential item functioning (DIF) in this item bank, i.e., the variation of item difficulty across different patient populations, which can be a relevant source of biased PRO scores. For the PROMIS physical function item bank, DIF has been found to be minor with regard to patient characteristics such as sex or country [32,33] but potentially prone to measurement bias related to age [33] or extremity (upper vs. lower) [28]. Whereas DIF related to the affected extremity (upper vs. lower) is rather obvious when assessing physical functioning, a more detailed analysis focusing on the specifically affected joint may be more beneficial.

However, to the best of our knowledge, there are no analyses investigating joint-related DIF. We think such analyses are important when assessing function in orthopedic patients because the affected joint strongly determines how various ADLs are affected (e.g., opening a tight jar is more difficult with an impaired hand than an impaired shoulder). This is likely to result in substantial DIF for a number of items when a general physical functioning item bank is employed.

We found DIF for the forgotten joint concept in total hip and total knee arthroplasty patients in a previous study [12] in which, for example, the item on getting up from a low sitting position showed substantially different item difficulty in hip and knee patients. Therefore joint-specific CAT measures may result in better measurement precision, as they do not suffer from this problem.

As limitations of our study we would like to note that our literature search did not include all available databases potentially relevant to shoulder outcome assessment, and we did not screen the references of the identified articles for further relevant articles or shoulder scores. However, the aim of our study was not to perform an exhaustive review of PRO instruments in shoulder patients, but to identify a large number of relevant issues. We believe that our initial list of 805 issues (243 unique issues relevant to joint awareness) is sufficient for developing a comprehensive item bank. Given the high level of redundancy in our initial issue list, it is unlikely that a more extensive literature search would have generated a substantial number of additional relevant items. Because item banks for physical functioning and related constructs often show high unidimensionality [12,24,26], we expect that a large proportion of our current set of 70 items can be included in a unidimensional IRT model. This will allow the set-up of a comprehensive item bank for conducting CAT assessments or for creating static short forms.

The next step in this process is the development of an IRT model to determine the psychometric characteristics of our item list; to achieve this we plan to recruit a large international sample of shoulder patients. This sample will comprise of different shoulder pathology groups (e.g. conservative and operative management of proximal humerus fractures, instability, rotator cuff tears, subacromial impingement and shoulder arthroplasty to investigate if there is differential item functioning depending on the different pathologies.

Subsequently we will validate the item bank using known-group comparisons and determine convergent validity through comparison with other well-established shoulder PRO measures (e.g. Shoulder Pain and Disability Index, Disability of the Arm, Shoulder and Hand). Furthermore, we will investigate responsiveness to change, and set up general population norms to facilitate interpretation of this new measure. Computer-adaptive scores based on a comprehensive item list and an IRT model offer superior measurement properties compared to traditional questionnaires as they adjust to the current condition of the individual patient. As tablet PCs are increasingly popular and available, we believe that PRO assessment in outpatient clinic using computer-adaptive questionnaires on tablet PCs offer a very appealing and efficient way of collecting PRO data. The advantages of individually tailored questionnaires are improved measurement precision and reduced patient burden.

## Conclusion

Based on literature search, expert opinion and patient feedback we created a list of 70 items for assessing joint awareness in shoulder patients. This item list was positively evaluated by 60 patients from Switzerland and the UK. This thorough methodological groundwork and the upcoming psychometric analyses will result in a novel measurement instrument, the Forgotten Joint Score – Shoulder. This new joint-specific PRO measure will allow the assessment of joint awareness after conservative or operative management of shoulder conditions.

## Abbreviations

AAOS: American Association of Orthopedic Surgeons
ADL: Activities of daily living
CAT: Computer-adaptive testing
DIF: Differential item functioning
EORTC: European Organisation for Research and Treatment of Cancer
FDA: Food and Drug Administration
FJS: Forgotten joint score
IRT: Item response theory
PRO: Patient-reported outcome
PROMIS: Patient-reported outcome measurement information system
SD: Standard deviation
